# Ionizable Lipid Nanoparticles Enhanced the Synergistic Adjuvant Effect of CpG ODNs and QS21 in a Varicella Zoster Virus Glycoprotein E Subunit Vaccine

**DOI:** 10.3390/pharmaceutics14050973

**Published:** 2022-04-30

**Authors:** Ning Luan, Han Cao, Yunfei Wang, Kangyang Lin, Cunbao Liu

**Affiliations:** Yunnan Key Laboratory of Vaccine Research and Development on Severe Infectious Diseases, Institute of Medical Biology, Chinese Academy of Medical Sciences and Peking Union Medical College, Kunming 650118, China; luanning@imbcams.com.cn (N.L.); caohan@imbcams.com.cn (H.C.); wangyf@imbcams.com.cn (Y.W.); linky@student.pumc.edu.cn (K.L.)

**Keywords:** varicella zoster virus, subunit vaccine, adjuvant, lipid nanoparticle, CpG ODNs (CpG oligodeoxynucleotides), QS21, humoral immunity, cell-mediated immunity

## Abstract

Varicella zoster virus (VZV) causes two diseases: varicella upon primary infection and herpes zoster when latent viruses in the sensory ganglia reactivate. While varicella vaccines depend on humoral immunity to prevent VZV infection, cell-mediated immunity (CMI), which plays a therapeutic role in the control or elimination of reactivated VZV in infected cells, is decisive for zoster vaccine efficacy. As one of the most abundant glycoproteins of VZV, conserved glycoprotein E (gE) is essential for viral replication and transmission between ganglion cells, thus making it an ideal target subunit vaccine antigen; gE has been successfully used in the herpes zoster vaccine Shingrix^TM^ on the market. In this report, we found that ionizable lipid nanoparticles (LNPs) approved by the Food and Drug Administration (FDA) as vectors for coronavirus disease 2019 (COVID-19) mRNA vaccines could enhance the synergistic adjuvant effect of CpG oligodeoxynucleotides (CpG ODNs) and QS21 on VZV-gE, affecting both humoral immunity and CMI. Vaccines made with these LNPs showed promise as varicella vaccines without a potential risk of herpes zoster, which identifies them as a novel type of herpes zoster vaccine similar to Shingrix^TM^. All of the components in this LNP-CpG-QS21 adjuvant system were proven to be safe after mass vaccination, and the high proportion of cholesterol contained in the LNPs was helpful for limiting the cytotoxicity induced by QS21, which may lead to the development of a novel herpes zoster subunit vaccine for clinical application.

## 1. Introduction

Subunit vaccines meet priorities including efficacy and safety compared to live attenuated vaccines for certain pathogens. Live attenuated varicella zoster virus (VZV) (e.g., the Oka strain developed by Takahashi in 1974 and approved by the Food and Drug Administration (FDA) in 1995) is the only vaccine on the market to prevent wild-type VZV infection, which causes varicella/chickenpox, yet the live virus in this vaccine can remain dormant in the sensory ganglia and reactivate as a wild-type strain, which may cause zoster/shingles with similar chances following immune system senescence or compromise [1]. Correspondingly, two forms of zoster vaccines were developed to boost the preexisting cell-mediated immunity (CMI) that was induced by primary wild-type VZV exposure or varicella vaccination, with the aim of playing a therapeutic role in the control or elimination of reactivated VZV in infected cells. A single subcutaneous dose of ZOSTVAX^®^ (developed by Merck & Co., Inc., Kenilworth, NJ, USA, and approved by the FDA in 2005) containing 19,400 plaque-forming units (PFUs) of attenuated virus can achieve 70% protection in people aged 50–59 but only 38% protection in people older than 70 years [2]. In addition, the difficulties related to virus purification and the strict conditions required for vaccine storage and shipping need to be addressed [3]. In contrast, two intramuscular doses of Shingrix^TM^ (developed by GlaxoSmithKline (GSK), Rockville, MD, USA, and approved by the FDA in 2017) have shown protective rates of approximately 90% against herpes zoster and postherpetic neuralgia, including in people older than 80 years [4,5]. In addition, this vaccine showed good efficacy and safety in immunocompromised patients, who are susceptible to herpes zoster [6,7,8,9,10,11].

The key to the success of subunit vaccines lies in the selection of effective antigens and the induction of appropriate adaptive immune responses. Unfortunately, immunization with a purified antigen alone is usually insufficient for proper immune induction and therefore requires the coadministration of immunostimulatory components called adjuvants. A further challenge for shingles subunit vaccines is the need to induce CMI, which plays a therapeutic role, rather than humoral immunity, which plays a preventive role, to restrict latent VZV and prevent zoster, which excludes the consideration of aluminum as an adjuvant [12,13,14,15,16,17]. Shingrix^TM^ includes glycoprotein E (gE) of VZV as its antigen because gE is one of the most abundant glycoproteins containing potential neutralizing epitopes and T-cell epitopes and is essential for viral replication and transmission between ganglion cells [18,19,20,21,22,23,24,25]. QS21 and monophosphoryl lipid (MPL) A have been shown to act synergistically to induce a high frequency of gE-specific CD4^+^ T cells in the liposome-based AS01B adjuvant system [26].

Oligodeoxynucleotides containing CpG motifs (CpG ODNs), which consist of a central unmethylated CG dimer present at a high frequency in bacterial and viral DNA, is recognized by mammalian Toll-like receptor (TLR) 9 and induces Th1 immune responses [27]. Although CpG ODNs have been approved by the FDA for use in a hepatitis B subunit vaccine as an adjuvant, doses up to 3 mg per injection increase not only costs but also the risk of side effects that are attributed to the systemic diffusion of this proinflammatory material [28,29,30]. In our previous reports, we proved that encapsulation of CpG ODNs into nanoparticles, including ionizable lipid nanoparticles (LNPs) that were proved to show adjuvant effects for protein subunit vaccines and approved by the FDA as vectors for coronavirus disease 2019 (COVID-19) mRNA vaccines, showed excellent efficacy in enhancing the antigen-specific CMI responses induced by subunit vaccines with dose sparing [31,32,33,34,35]. In other previous reports, synergy between QS21 and CpG ODNs in cancer and hepatitis C subunit vaccines was shown to enhance CMI [36,37]. Considering that the LNP system contains cholesterol, which is necessary to quench the hemolytic activity of QS21, the LNP-CpG-QS21 adjuvant system reported in this study may pave the way for the development of a novel herpes zoster subunit vaccine for clinical application [38,39,40,41].

## 2. Materials and Methods

### 2.1. Vaccine Preparation and Characterization

The components of the prepared vaccines are shown in Table 1 and Table 2.

The extracellular domain of VZV-gE ((AtaGenix Laboratory Co., Ltd., Wuhan, China) was expressed by Chinese hamster ovary (CHO) cells and dissolved in phosphate-buffered saline (PBS, pH 7.4) before use. Imject^TM^ Alum and QS21 (*Quillaja saponaria*, QS) were purchased from Thermo Scientific (Rockford, IL, USA) and Alpha Diagnostic (San Antonio, TX, USA), respectively. Phosphorothioated CpG ODN products including BW006 (5′-tcg acg ttc gtc gtt cgt cgt tc-3′) and 2395 (5′-tcg tcg ttt tcg gcg cgc gcc g-3′) were synthesized by Sangon Biotech (Shanghai, China).

For the groups in Table 1 (BW006+2395, QS21, BW006+2395+QS21 (B2Q) and Alum groups) and the B2Q and B2Q-Alum groups in Table 2, vaccines were formulated by mixing the agents thoroughly in PBS before immunization. For vaccines containing alum, the volume ratio of alum to the other vaccine constituent was 1:1.

For the B2Q-LNP and B2 (BW006+2395)-LNP group, vaccines were prepared using a modified version of the procedure described in our previous report [31]. Briefly, lipids (from AVT Pharmaceutical Technology Co., Ltd., Shanghai, China) were dissolved in ethanol at molar ratios of ionizable lipids (Dlin-MC3-DMA, CAS No. 1224606-06-7): DSPC: cholesterol: DMG-PEG2000 = 50:10:38.5:1.5. The protein gE, CpG ODNs and QS21 were dissolved in 100 mM citrate buffer (pH 4.0), completely mixed and combined with lipid mixtures at a ratio of 3:1 with a microfluidic mixer (Precision Nanosystems, Vancouver, BC, Canada). The formulations were dialyzed against PBS and concentrated with a centrifugal filtration tube (Millipore, Carrigtwohill, Ireland, MW 10000, 0.22 µm). The diameter and polydispersity index (PDI) of particles were tested with a Zetasizer Nano ZS particle size analyzer (Malvern Panalytical, Malvern, UK).

The formulation was lysed with 0.1 M NaOH containing 0.1% SDS (*m*/*v*) at room temperature overnight. The loaded gE was detected with a bicinchoninic acid (BCA) protein assay kit (Beyotime, Shanghai, China). The loaded nucleic acid adjuvants were detected with a Quant-iT OliGreen ssDNA Reagent Kit (Thermo Fisher, Eugene, OR, USA). With free QS21 as the standard, loaded QS21 was detected by high performance liquid chromatography (HPLC) with an inside diameter of 4.6 mm × length 250 mm C18 column (Waters, Ireland) and the flow gradient as follows: H_2_O + 0.1% (*v*/*v*) trifluoroacetic acid (TFA) as eluent A and acetonitrile +0.1% TFA as eluent B, 5% eluent B in 20 min, 6–40% eluent B in 21–27 min, 41–60% eluent B in 28–47 min, 100% eluent B for 10 min, and back to 5%. Flow rate was set as 1 mL/min by Waters 1525 Binary HPLC Pump, and UV at 205 nm was detected by Waters 2998 Photodiode Array Detector [42].

### 2.2. Hemolytic Assay

Blood samples from specific pathogen-free (SPF) C57BL/6J mice (female, 6–8 weeks old, 16–18 g, supplied by the Central Service of the Institute of Medical Biology, Chinese Academy of Medical Sciences (IMB, CAMS)) were mixed with sterilized Alsever’s solution (8.0 g/L citrate sodium, 20.48 g/L glucose, 0.548 g/L citric acid, 4.2 g/L NaCl, pH 6.1) at a volume ratio of 1:1, centrifuged at 1000× *g* for 5 min, and washed three times with 0.9% (*w*/*v*) saline. Erythrocytes were diluted with 0.9% saline to a concentration of 10^7^ cells/mL. When 10% Triton X-100 (*v*/*v*) dissolved in saline was used as the positive control and the same volume of saline was used as the blank control, serial diluted samples (75 µL) were incubated with 175 µL diluted erythrocytes at 37 °C for 30 min. The supernatants were gathered by centrifuge at 1000× *g* for 5 min, and absorbance at 540 nm was measured. The hemolysis rate was calculated by [(Absorbance _sample_ − Absorbance _blank control_)/(Absorbance _positive control_ − Absorbance _blank control_)] × 100%.

### 2.3. Mouse Studies

Female SPF C57BL/6J mice (6–8 weeks old, 20–22 g, from the Central Service of IMB, CAMS) were divided randomly into 9 groups with 6 mice in each group (N = 6). The mice were immunized intramuscularly in the tibialis muscle two times with 50 μL of immunogen at 4-week intervals. Whole-blood samples and spleen cells were collected 2 weeks after the final immunization as described elsewhere [43]. Sera were obtained after centrifugation at 1000× *g* for 30 min and stored at −80 °C before use.

### 2.4. Enzyme-Linked Immunosorbent Assay (ELISA)

A recombinant gE protein (2 μg/mL) was used to precoat 96-well microplates overnight at 4 °C and then washed with 0.05% (*v*/*v*) polysorbate 20 in PBS (PBST) 3 times before the addition of 5% (*w*/*v*) skim milk in PBS for one hour to block the plates. Then, twofold-diluted mouse serum samples in 1% skim milk were incubated in the plates for 1 h. Goat anti-mouse IgG conjugated with horseradish peroxidase (HRP) (1:10,000. Bio–Rad, Hercules, CA, USA) was used as the detection antibody. Five minutes after the addition of the mixed substrate 3,3,5,5-tetramethylbenzidine (TMB; BD, San Diego, CA, USA), the reaction was stopped by addition of 1 M sulfuric acid. Optical density at 450 nm was detected with a spectrophotometer (BioTek Instruments, Inc., Winooski, VT, USA). IgG titers were defined as the maximum dilution multiple showing cutoff signals above OD450 = 0.15. According to this definition, antibody titers of serum show OD450 lower than 0.15 at a dilution of 1:2000 were defined as 100 for statistical calculations.

### 2.5. Cytokine Analysis

Splenocytes were suspended in 1640 medium supplemented with 10% (*v*/*v*) fetal bovine serum (FBS) and penicillin–streptomycin (Thermo Fisher) at a final concentration of 1 × 10^7^ cells/mL. Then, 1 × 10^6^ cells were added to each well of a 96-well plate (Corning Inc., Corning, NY, USA). With 10 μL of PMA + ionomycin (stock solution concentrations: 500 ng/mL + 10 μg/mL; DAKEWE, Beijing, China) as positive control, gE was added to each well at a final concentration of 10 μg/mL. After incubation at 37 °C for 24 h in a 5% CO_2_ atmosphere, the supernatants of the cells were collected to test the contents of IL-2 and IFN-γ. Briefly, anti-IL-2 (3 μg/mL) and anti-IFN-γ (4 μg/mL) capture antibodies (Invitrogen, Carlsbad, CA, USA) dissolved in PBS were added to coat 96-well plates and incubated overnight at 4 °C. After blocking with 5% (*w*/*v*) skim milk at 37 °C for 1 h, 50 μL of cell supernatant was added to each well and incubated for another 3 h at room temperature. PBS-dissolved standard mouse IL-2 and IFN-γ proteins (PeproTech, Cranbury, NJ, USA) were used to generate standard curves. Biotin-conjugated detection antibodies specific for IL-2 or IFN-γ (2 μg/mL, Invitrogen, USA) and HRP-conjugated streptavidin (1 μg/mL, BioLegend, San Diego, CA, USA) were then added and incubated for 1.5 h. The results were detected as mentioned above in the ELISA analysis.

### 2.6. Enzyme-Linked Immunospot (ELISPOT) Assay

An ELISPOT assay was performed to analyze IL-2 and IFN-γ production by using an ELISPOT assay kit (BD) according to the manufacturer’s instructions. Briefly, splenocytes from immunized mice were seeded in 96-well plates, and the final concentrations of splenocytes from mice immunized as shown in Table 1 and Table 2 were 5 × 10^5^ cells/well and 2 × 10^5^ cells/well, respectively. The protein gE was used at a final concentration of 20 µg/mL to stimulate gE-specific T-cell responses for 16 h, and the same volumes of medium or PMA + ionomycin were used as the negative control and positive control, respectively. After removal of the cell supernatants, spots were developed by an ALL-IN-ONE mouse ELISPOT Accessory kit and counted with an ELISPOT reader system (Autoimmun Diagnostika GmbH, Strassberg, Germany) [33].

### 2.7. Flow Cytometry

Flow cytometry reagents were all supplied by BioLegend (San Diego, CA, USA). 1 × 10^6^ splenocytes were incubated with 10 µg/mL protein gE at 37 °C in 5% CO_2_ for 2 h to stimulate the expression of antigen-specific cytokines, and 5 µg/mL brefeldin A was then added and incubated overnight under the same conditions to block cytokine release. After washing with staining buffer, 100 μL of Zombie NIR™ was added to each well and incubated for 30 min to determine the proportion of dead cells in the sample. Then, 5 µg/mL anti-CD16/CD32 antibodies was added and incubated at 4 °C for 10 min to block nonspecific binding to Fc receptors. PerCP-Cy5.5-conjugated anti-mouse CD4, FITC-conjugated anti-mouse CD8, BV510-conjugated anti-mouse CD44, and BV421-conjugated anti-mouse CD62L antibodies were subsequently added and incubated for another 30 min at 4 °C for surface maker staining. PE-conjugated anti-mouse IFN-γ and APC-conjugated anti-mouse IL-2 antibodies were used for intracellular staining. After staining, live cells were gated (forward and side scatter, FSC/SSC), and acquisition with more than 20,000 events for CD4^+^ or CD8^+^ T cells in each sample were analyzed with a CytoFLEX flow cytometer (Beckman, Indianapolis, IN, USA) and FlowJo_V10 software (BD, Franklin Lakes, NJ, USA).

### 2.8. Statistical Analysis

Data were analyzed with GraphPad Prism 9.2 (GraphPad Software Inc., La Jolla, CA, USA) and are expressed as the mean ± standard deviation (SD). Significant differences among experimental groups were analyzed by one-way analysis of variance (ANOVA) followed by Dunnett’s multiple comparisons test. Asterisks represent the *p* value classification: * *p* ≤ 0.05, ** *p* ≤ 0.01, and *** *p* ≤ 0.001.

## 3. Results

### 3.1. CpG ODNs and QS21 Act Synergistically to Enhance VZV-gE-Specific CMI but Not the Humoral Response

**Figure 1:**
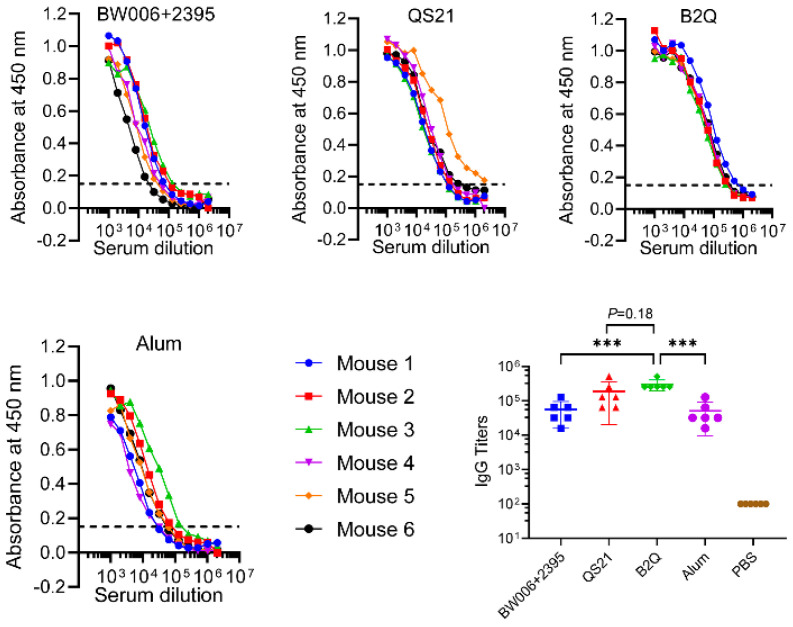
Humoral responses of mice in the adjuvant synergy study. For enzyme-linked immunosorbent assay (ELISA) analysis of each serum sample from immunized mice, the horizontal coordinate is the fold change in the serum dilution, and the vertical coordinate is the absorbance value measured at 450 nm. The dashed lines (OD 450 = 0.15) were set as the cutoff signal for the values of IgG titers. For statistical analysis, each dot represents data from a single mouse, N = 6. Data are shown as the mean ± standard deviation (SD) and were analyzed by one-way analysis of variance (ANOVA), with the mean of the B2Q group used as a control. *** *p* ≤ 0.001.

The gE-specific IgG titer was 298,667 in B2Q (adjuvanted with CpG BW006+2395 and QS21)-immunized mouse serum, which was the highest titer among all the vaccines designed for the adjuvant synergy study (Figure 1). This titer was 5.3 times greater than that of the CpG alone-adjuvanted group (group BW006+2395, IgG titer of 56,000, *p* < 0.001), 1.6 times greater than that of the QS21 alone-adjuvanted group (group QS21, IgG titer of 192,000, *p* = 0.18), and 5.9 times greater than that of the alum-adjuvanted group (Alum group, *p* < 0.001). The difference between the B2Q group and the QS21 group was not significant (*p* = 0.18), which was defined as no synergistic effect.

**Figure 2:**
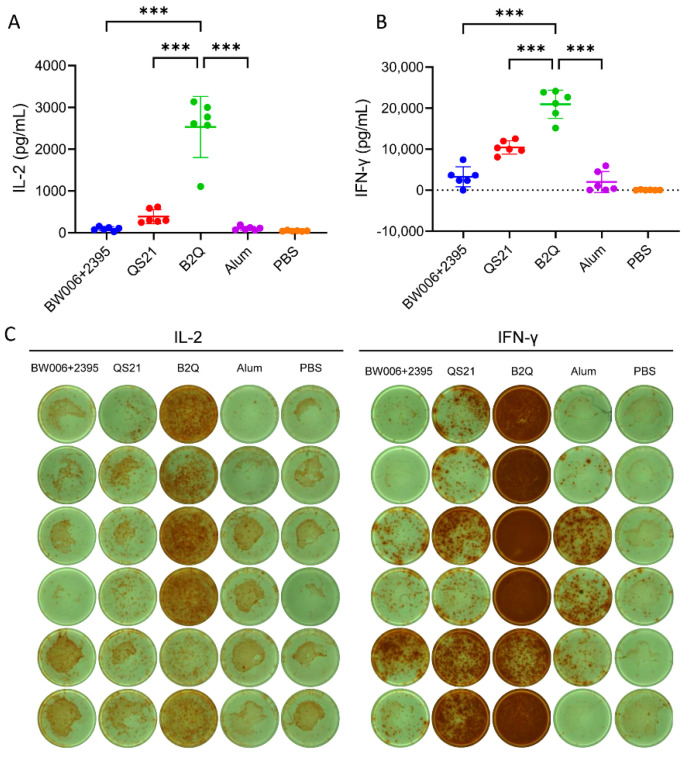
ELISA and ELISPOT analyses performed with splenocytes isolated from immunized mice in the adjuvant synergy study. The concentrations of IL-2 (**A**) and IFN-γ (**B**) secreted by splenocytes upon stimulation with 10 μg/mL protein gE were measured by ELISA. N= 6, each dot represents an independent mouse. Data are shown as the mean and SD and were analyzed by ANOVA, with the mean of the B2Q group used as a control. (**C**) IL-2- and IFN-γ-producing splenocytes (per 5 × 10^5^ cells) induced by stimulation with 20 μg/mL protein gE were detected by ELISPOT. Images for all immunized mice are presented. N = 6. *** *p* ≤ 0.001.

Significant synergistic effects between CpG ODNs and QS21 were detected for CMI (Figure 2). By ELISA, the IL-2 level in the supernatant of the B2Q-adjuvanted group was 2532 pg/mL (Figure 2A). This concentration was 26.9 times greater than that of the CpG alone-adjuvanted group (94.03 pg/mL, *p* < 0.001) and 6.6 times greater than that of the QS21 alone-adjuvanted group (384.4 pg/mL, *p* < 0.001). The IFN-γ level in the supernatant of the B2Q-adjuvanted group was 20952 pg/mL (Figure 2B). This concentration was 6.5 times greater than that of the CpG alone-adjuvanted group (3245 pg/mL, *p* < 0.001) and 2 times greater than that of the QS21 alone-adjuvanted group (10417 pg/mL, *p* < 0.001). These synergistic effects were more obvious in the results of the ELISPOT assay. When splenocytes were seeded at 5 × 10^5^ cells/well, the cytokine-secreting cells in the B2Q-adjuvanted group were so numerous they could not be counted (Figure 2C).

### 3.2. CpG ODNs and QS21 Act Synergistically to Induce VZV-gE-Specific CD4^+^ but Not CD8^+^ T Cells

**Figure 3:**
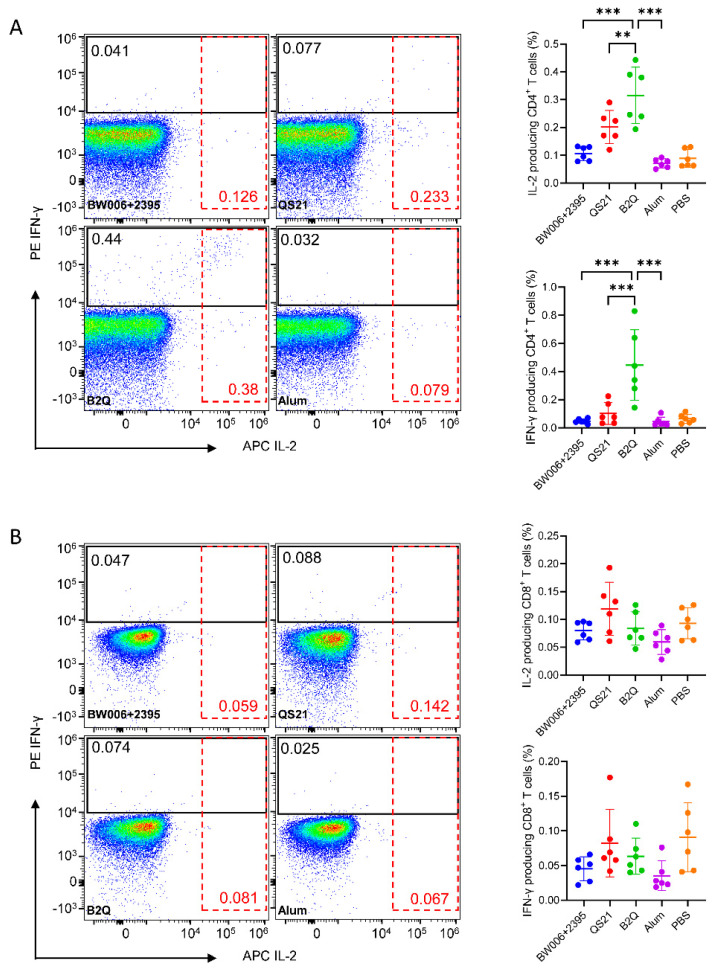
Flow cytometry assay assessing gE-specific cytokine-producing CD4^+^ (**A**) and CD8^+^ (**B**) T cells in mice in the adjuvant synergy study. Splenocytes from immunized mice were treated with 10 μg/mL protein gE, and after blocking with brefeldin A, IL-2- and IFN-γ-producing CD4^+^ (**A**) or CD8^+^ (**B**) T cells were detected. Pseudocolor images displaying representative results near the average value are presented in the left panel. N = 6, points represent individual mice. Data are shown as the mean and SD and were analyzed by ANOVA, with the mean of the B2Q group used as a control. ** *p* ≤ 0.01; *** *p* ≤ 0.001.

According to flow cytometric analysis (Figure 3A), the proportion of IL-2-expressing CD4^+^ T cells after gE stimulation in the B2Q-adjuvanted group was 0.3155%. This level was 3 times greater than that of the CpG alone-adjuvanted group (0.1067%, *p* < 0.001) and 1.6 times greater than that of the QS21 alone-adjuvanted group (0.2013%, *p* = 0.006). The proportion of IFN-γ-expressing CD4^+^ T cells after gE stimulation in the B2Q-adjuvanted group was 0.4457%. This level was 9.3 times greater than that of the CpG alone-adjuvanted group (0.04767%, *p* < 0.001) and 4.3 times greater than that of the QS21 alone-adjuvanted group (0.1025%, *p <* 0.001). In contrast, no VZV-gE-specific CD8^+^ T cells were induced after immunization with any of the designed vaccines, with the IL-2- and IFN-γ-producing CD8^+^ T-cell proportions in the vaccinated groups being comparable to those in the PBS-immunized control group by flow cytometric analysis (Figure 3B).

### 3.3. LNPs Efficiently Encapsulated gE, CpG ODNs and QS21 with a Uniform Particle Size

**Figure 4:**
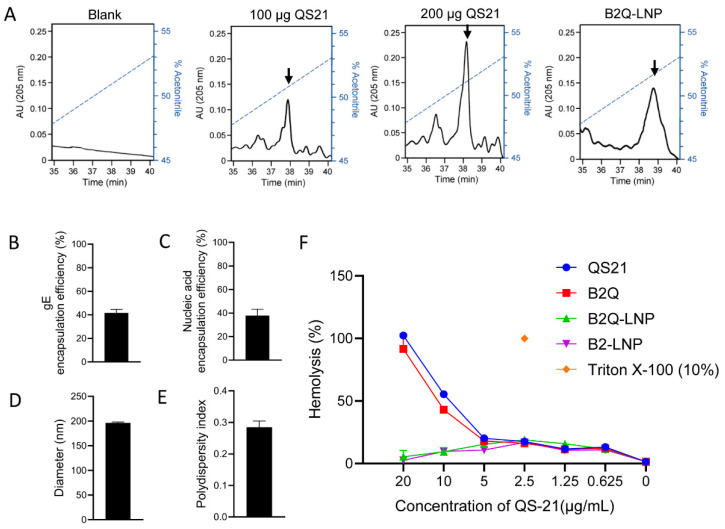
Characterization of the lipid nanoparticle (LNP)-CpG-QS21 adjuvanted vaccine and hemolytic assay. (**A**) The encapsulation efficiency analysis of QS21 by high performance liquid chromatography. Flow gradient is shown with dotted lines and eluted QS21 is indicated by arrows. (**B**) The encapsulation efficiency of protein gE. (**C**) The encapsulation efficiency of CpG ODNs. (**D**) diameter. (**E**) polydispersity index. (**F**) hemolysis analysis. Experiments were repeated three times, and the data are shown as the mean ± SD.

When 200 µg of QS21 was fed for LNP encapsulation, approximately 100–160 µg was detected by HPLC analysis (Figure 4A), which resulted in 50–80% of encapsulation efficiency and 4–6 μg of QS21 in each dose of prepared LNPs, comparable to 5 μg per dose in the other mixed vaccines. A total of 12 µg of gE was included in each dose of the LNP vaccine. The average encapsulation efficiency of the gE protein was 41.64% (Figure 4B), which resulted in 5 µg of gE in each dose of prepared LNPs, which was half that in the other mixed vaccines. A total of 15 µg of CpG ODNs was added to each dose of the LNP vaccine. The mean value of the CpG ODN encapsulation efficiency was 37.71% (Figure 4C), which resulted in 5.66 μg of CpG ODNs in each dose of prepared LNPs compared to 10 µg per dose in the other mixed vaccines. The average LNP diameter was 196.1 nm (Figure 4D), with good uniformity, showing a low PDI (a measure of the heterogeneity of a sample based on size) of 0.28 (Figure 4E).

### 3.4. LNPs Quenched the Hemolytic Activity of QS21

Nearly 100% erythrocytes were lysed with 20 µg/mL free QS21 with or without CpG ODNs (QS21 or B2Q in Figure 4F). When encapsulated with LNPs (B2Q-LNP in Figure 4F), QS21 at the same concentration showed hemolytic activity as low as that comparable to the B2-LNP group without QS21 (hemolysis rate at approximately 2.6%), which indicates quench of hemolytic activity.

### 3.5. LNPs Enhanced the Synergistic Adjuvant Effect of CpG ODNs and QS21 on VZV-gE-Specific Humoral Responses

**Figure 5:**
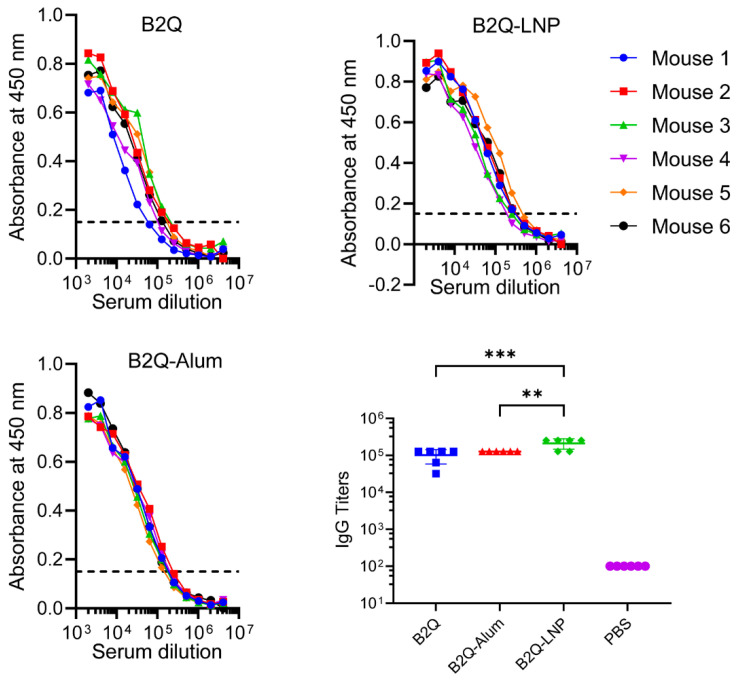
Humoral responses of mice in the LNP encapsulation study. For ELISA analysis of each serum sample from immunized mice, the horizontal coordinate is the fold change in the serum dilution, and the vertical coordinate is the absorbance value measured at 450 nm. The dashed lines (OD 450 = 0.15) were set as the cutoff signal for the values of IgG titers. For statistical analysis, each dot represents data from a single mouse, N = 6. Data are shown as the mean ± SD and were analyzed by ANOVA, with the mean of the B2Q-LNP group used as a control. ** *p* ≤ 0.01; *** *p* ≤ 0.001.

The gE-specific IgG titer was 213,333 in the B2Q-LNP-immunized mouse serum, which was the highest titer among those induced by all the vaccines designed for the LNP encapsulation study (Figure 5). It was 2.1 times greater than that of the unencapsulated B2Q group (IgG titer of 101,333, *p* < 0.001) and 1.7 times greater than that of the B2Q-Alum-adjuvanted group (IgG titer of 128,000, *p* = 0.003). The differences between the B2Q-LNP group and the other groups were all significant and were therefore defined as synergistic effects.

### 3.6. LNPs Enhanced the Synergistic Adjuvant Effect of CpG ODNs and QS21 on VZV-gE-Specific CD4^+^ but Not CD8^+^ CMI

**Figure 6:**
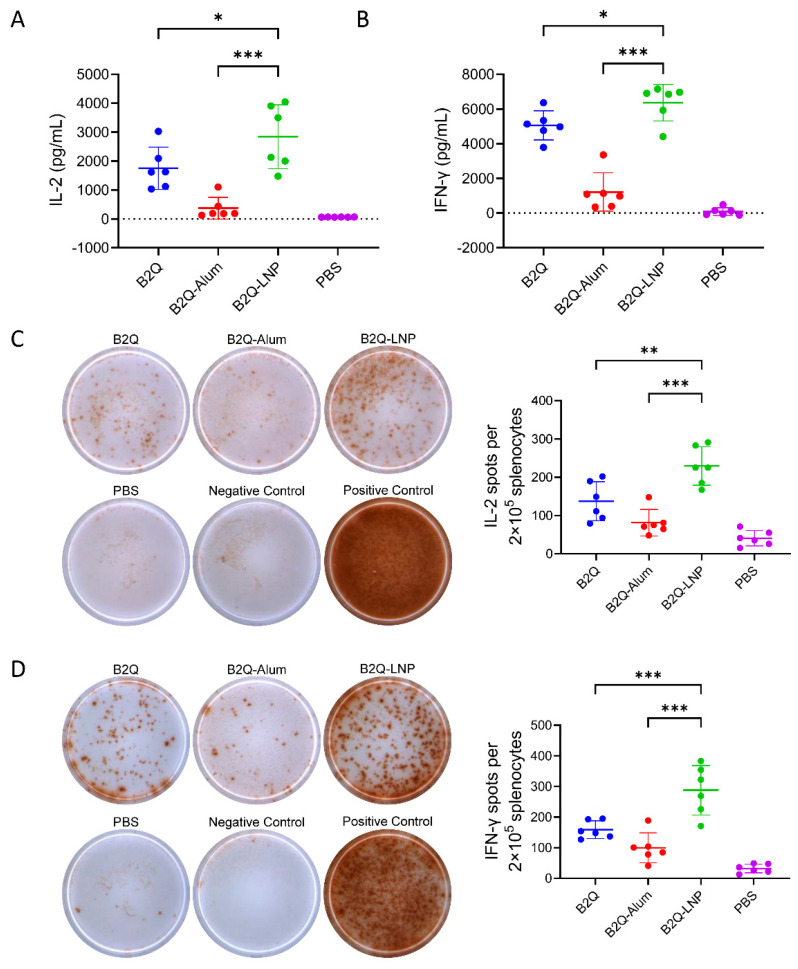
ELISA and ELISPOT analyses performed with splenocytes isolated from immunized mice in the LNP encapsulation study. The concentrations of IL-2 (**A**) and IFN-γ (**B**) secreted by splenocytes upon stimulation with 10 μg/mL protein gE were measured by ELISA. (**C**) IL-2- and (**D**) IFN-γ-producing splenocytes (per 2 × 10^5^ cells) induced by stimulation with 20 μg/mL protein gE were detected by ELISPOT. Images displaying representative results near the average value are presented in the left panel. Cells treated with the same volume of medium or PMA+ionomycin were used as the negative control and positive control, respectively. N = 6, points represent individual mice. Data are shown as the mean and SD and were analyzed by ANOVA, with the mean of the B2Q-LNP group used as a control. * *p* ≤ 0.05; ** *p* ≤ 0.01; *** *p* ≤ 0.001.

LNP encapsulation enhanced the synergistic adjuvant effect of CpG ODNs and QS21 on VZV-gE-specific CMI (Figure 6). By ELISA analysis, the IL-2 level was 2843 pg/mL in the supernatant of the B2Q-LNP-adjuvanted group (Figure 6A). This level was 1.6 times greater than that of the B2Q-adjuvanted group (1752 pg/mL, *p* = 0.04) and 7.7 times greater than that of the B2Q-Alum-adjuvanted group (368.6 pg/mL, *p* < 0.001). The IFN-γ level was 6366 pg/mL in the supernatant of the B2Q-LNP-adjuvanted group (Figure 6B). This level was 1.3 times greater than that of the B2Q-adjuvanted group (5059 pg/mL, *p* = 0.05) and 5.2 times greater than that of the B2Q-Alum-adjuvanted group (1216 pg/mL, *p* < 0.001). By ELISPOT analysis, the number of IL-2-secreting cells after gE stimulation in the B2Q-LNP adjuvanted group was 229.3 per 2 × 10^5^ splenocytes (Figure 6C). This number was 1.7 times greater than that of the B2Q-adjuvanted group (137.5 per 2 × 10^5^ splenocytes, *p* = 0.03) and 2.8 times greater than that of the B2Q-Alum-adjuvanted group (81.33 per 2 × 10^5^ splenocytes, *p* < 0.001). The number of IFN-γ-secreting cells after gE stimulation in the B2Q-LNP-adjuvanted group was 287.5 per 2 × 10^5^ splenocytes (Figure 6D). This number was 1.8 times greater than that of the B2Q-adjuvanted group (159 per 2 × 10^5^ splenocytes, *p* < 0.001) and 2.9 times greater than that of the B2Q-Alum-adjuvanted group (99.67 per 2 × 10^5^ splenocytes, *p* < 0.001).

**Figure 7:**
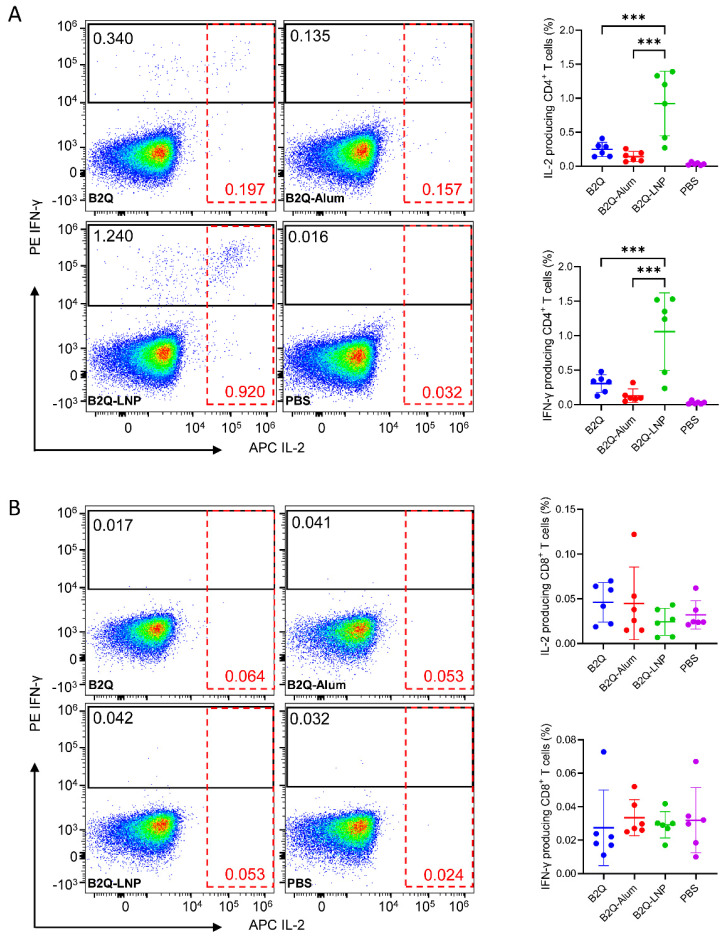
Flow cytometry assay assessing cytokine-producing gE-specific CD4^+^ (**A**) and CD8^+^ (**B**) T cells in mice in the LNP encapsulation study. Splenocytes from immunized mice were treated with 10 μg/mL protein gE, and after blocking with brefeldin A, IL-2- and IFN-γ-producing CD4^+^ (**A**) or CD8^+^ (**B**) T cells were detected. Pseudocolor images displaying representative results near the average value are presented in the left panel. N = 6, points represent individual mice. Data are shown as the mean and SD and were analyzed by ANOVA, with the mean of the B2Q-LNP group used as a control. *** *p* ≤ 0.001.

According to flow cytometric analysis (Figure 7A), the proportion of IL-2-expressing CD4^+^ T cells after gE stimulation in the B2Q-LNP-adjuvanted group was 0.9217%. This level was 3.7 times greater than that of the B2Q-adjuvanted group (0.2462%, *p* < 0.001) and 6.5 times greater than that of the B2Q-Alum-adjuvanted group (0.1422%, *p* < 0.001). The proportion of IFN-γ-expressing CD4^+^ T cells after gE stimulation in the B2Q-LNP-adjuvanted group was 1.058%. This level was 3.5 times greater than that of the B2Q-adjuvanted group (0.306%, *p* < 0.001) and 8.2 times greater than that of the B2Q-Alum-adjuvanted group (0.1298%, *p <* 0.001). In contrast, no VZV-gE-specific CD8^+^ T cells were induced after immunization with any of the designed vaccines, with the IL-2- and IFN-γ-producing CD8^+^ T-cell proportions in the vaccinated groups being comparable to those in the PBS-immunized control group by flow cytometric analysis (Figure 7B).

### 3.7. LNP-Encapsulated CpG ODNs and QS21 Induced gE-Specific CD4^+^ Memory T Cells

**Figure 8:**
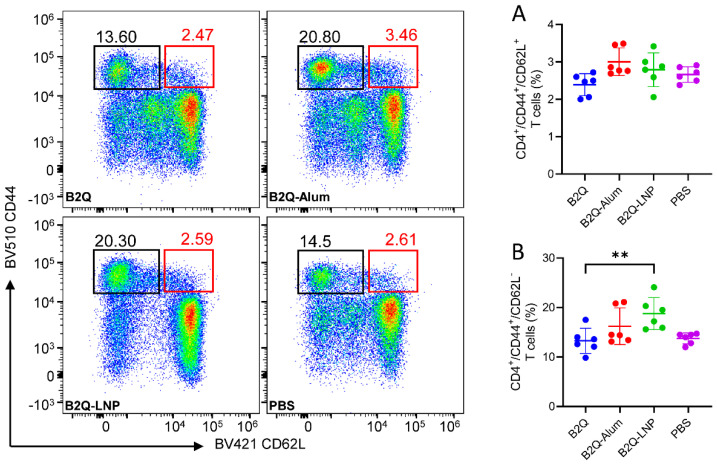
Flow cytometry assay assessing gE-specific CD4^+^ memory T cells in the LNP encapsulation study. Splenocytes from immunized mice were treated with 10 μg/mL protein gE, and after blocking with brefeldin A, memory CD4^+^ T cells were detected by CD44+/CD62L+ gating. (**A**) Proportion of central memory T cells. (**B**) Proportion of effector memory T cells. Pseudocolor images displaying representative results near the average value are presented in the left panel. N=6, points represent individual mice. Data are shown as the mean and SD and were analyzed by ANOVA, with the mean of the B2Q-LNP group used as a control. ** *p* ≤ 0.01.

According to flow cytometric analysis (Figure 8), the proportion of CD4^+^ central memory cells (CD44^+^CD62L^+^) was comparable between the B2Q-LNP-adjuvanted group and the other groups (Figure 8A). For the proportion of CD4^+^ effector memory cells (CD44^+^CD62L^−^, Figure 8B), the proportion was 18.77% in B2Q-LNP-immunized mouse splenocytes, which was the highest proportion among all those induced by the vaccines designed for the encapsulation study. This level was 1.4 times greater than that of the B2Q-adjuvanted group (13.28%, *p* = 0.008) and 1.2 times greater than that of the B2Q-Alum-adjuvanted group (16.22%, *p* = 0.3 without a significant difference).

## 4. Discussion

Until recently, aluminum salts, including aluminum hydroxide and aluminum hydroxyphosphate, were the only adjuvants licensed for human vaccines worldwide. These salts primarily induce humoral immunity typical of Th2 responses when subunit or inactivated vaccines are administered but inefficiently induce CMI, which is essential for vaccines targeting intracellular pathogens or tumors [44,45]. QS21 is a mixture of two isomeric saponins (65% QS21-Apiose and 35% QS21-Xylose) extracted from the bark of the evergreen tree *Q. saponaria* native to temperate central Chile. Its amphipathic structure endows QS21 with the capacity to disrupt cellular membranes, including endosome-lysosome destabilization, which facilitates antigen translocation into the cytosol for proteasome-independent cross-presentation by major histocompatibility complex (MHC) class I to induce CMI, instead of classic MHC II presentation inducing humoral responses [38,39,40,41]. Related to its membrane-disrupting characteristics, the cytotoxicity of QS21 can be quenched by formulations containing cholesterol (e.g., adjuvant systems, including the AS01 series and ISCOMATRIX), which reduce the affinity of its triterpene nucleus for cholesterol in cell membranes [26,46,47]. While the synergistic adjuvant effect of QS21 and CpG ODNs has been reported to enhance CMI induced by cancer and hepatitis C subunit vaccines, the absence of cholesterol in these combinations may hinder their clinical application due to safety concerns [36,37].

LNP systems contain a high proportion of cholesterol (38.5–42.7% molar ratios) and have been approved by the FDA for use in COVID-19 mRNA vaccines and proven to be safe after mass vaccination. In theory, the extracellular domain of VZV-gE as a hydrophilic protein could be effectively encapsulated by the LNP system like nucleic acid substances. In fact, the encapsulation efficiency of gE could be 100% when gE alone at the input of 15 µg/dose was administered into LNP, which is deduced to be encapsulating instead of co-administering for absorbance by LNP. We also noticed that the addition of other components including CpG and polyinosinic-polycytidylic acid (poly(I:C)) lowered the gE encapsulation efficiency to 80% [31]. The gE encapsulation efficiency in this LNP-CpG-QS21 adjuvant system seems to be lower than expected, which is only 41.64% when gE were at the input of 10 µg/dose. Except for the potential influence of of QS21 on either gE character or the wrapping mode of LNP, the LNP mixtures we used in this study were not freshly mixed. They were in fact cryopreserved for several months after dissolution and sub-package. We suspect that this process may affect the encapsulation ability of the LNPs. Because LNP systems are still new, we have not retrieved any relevant information on this aspect yet.

When used as a carrier in the LNP-CpG-QS21 adjuvant system, the LNP systems studied doubled (i.e., 2.1 times) the VZV-gE-specific humoral response induced by the combination of CpG and QS21 alone, indicating their superiority to alum as an adjuvant carrier (i.e., 1.7 times) for the combination of CpG and QS21 as immunostimulators. Considering that VZV-gE containing potential neutralizing epitopes and VZV-gE-specific antibodies could efficiently block VZV infection, this LNP-CpG-QS21 adjuvanted subunit vaccine may show promise as a safe varicella vaccine to prevent chickenpox and the risk of shingles derived from live attenuated VZV vaccination [24,31].

CMI, which plays a therapeutic role, rather than humoral immunity, which plays a preventive role, is decisive in restricting latent VZV and preventing zoster [12,13,14,15,16]. Among CMI indicators, gE-specific Th1 CD4^+^ T cells are studied more frequently than CD8^+^ T cells when evaluating the potential of herpes zoster vaccines in animal experiments and clinical trials because CD8^+^ T cells are always undetectable or present at low frequencies after VZV infection or vaccination [20,26,48,49,50,51]. According to our previous experiences, LNP alone could only elevate gE-specific CMI slightly by ELISPOT analysis [31]. From this point of view, the LNP-CpG-QS21 adjuvant systems studied tripled (3.5–3.7 times) the VZV-gE-specific Th1 CD4^+^ T cell population induced by the combination of CpG and QS21 alone. Interestingly, while alum, as an adjuvant carrier, slightly elevated the VZV-gE-specific humoral responses (from 101333 to 128000) induced by the combination of CpG and QS21 as immunostimulators, its addition as a carrier reduced the ability of the combination of CpG and QS21 as immunostimulators to induce antigen-specific CMI based on all of the indicators tested, which may be attributed to its Th2 characteristics.

In addition to the magnitude and type of adaptive immune responses induced, the duration of the immune response should be evaluated for vaccines. In mice, when naive T cells (CD44^low^CD62L^+^) were activated, they developed into central memory (CD44^high^CD62L^+^) and/or effector memory (CD44^high^CD62L^−^) T cells. While central memory skewing is observed in CD8^+^ T cells and effector memory skewing is observed in CD4^+^ T cells in the resting state, both the CD4^+^ and CD8^+^ T-cell subsets are predominantly of the effector memory phenotype in the peripheral organs [52,53,54,55,56]. Consistent with previous reports showing that nanoparticles are helpful in inducing CD4+ effector memory T cells, the addition of LNP carriers was helpful in elevating gE-specific CD4+ effector memory T cell levels compared with the combination of CpG and QS21 alone in our results [57,58].

## 5. Conclusions

In conclusion, our study showed that LNPs enhanced the synergistic adjuvant effect of CpG and QS21. The components of this LNP-CpG-QS21 adjuvant system have been proven to be safe after mass vaccination, and the high proportion of cholesterol contained in LNPs is helpful for quenching the cytotoxicity induced by QS21. Although the LNP alone and LNP mixed gE as controls should be added for further confirmation of the pattern of this LNP-CpG-QS21 adjuvant system wrapping gE, and the mechanisms of synergy between the components of this adjuvant system remains to be further clarified, the robust induced VZV-gE-specific humoral and CMI responses showed promise as varicella vaccines without the potential risk of zoster and as a novel type of zoster vaccine similar to Shingrix^TM^.

## Figures and Tables

**Table 1:** Composition of each dose of vaccine for the adjuvant synergy study.

Vaccine Group	gE (μg/dose)	Alum	BW006 (μg/dose)	2395 (μg/dose)	QS21 (μg/dose)
BW006+2395	10	-	5	5	-
QS21	10	-	-	-	5
B2Q	10	-	5	5	5
Alum	10	√	-	-	-
PBS	-	-	-	-	-

- Not added; √ Added.

**Table 2:** Feeding components of each dose of vaccine for the encapsulation study.

Vaccine Group	gE (μg/dose)	Alum	BW006 (μg/dose)	2395 (μg/dose)	QS21 (μg/dose)
B2Q	10	-	5	5	5
B2Q-Alum	10	√	5	5	5
B2Q-LNP	12	-	7.5	7.5	7.5
B2-LNP	12		7.5	7.5	
PBS	-	-	-	-	-

- Not added; √ Added.

## Data Availability

All the data from the study are available from the corresponding author upon reasonable request.
